# Handgrip strength: a reliable predictor of postoperative early ambulation capacity for the elderly with hip fracture

**DOI:** 10.1186/s12891-021-03964-9

**Published:** 2021-01-22

**Authors:** Chih-Mai Chang, Cheng-Hung Lee, Cheng-Min Shih, Shun-Ping Wang, Yung-Cheng Chiu, Cheng-En Hsu

**Affiliations:** 1 Department of Orthopedics, Taichung Veterans General Hospital, Taichung, Taiwan; 2 Department of Food Science and Technology, Hung Kuang University, Taichung, Taiwan; 3 Institute of Biomedical Engineering, National Chiao Tung University, Hsinchu, Taiwan; 4 Department of Physical therapy, Tunghai University, Taichung, Taiwan; 5 Sports Recreation and Health Management Continuing Studies-Bachelor’s Degree Completion Program, Tunghai University, Taichung, Taiwan; 6 Department of Orthopedic Surgery, China Medical University, Taichung, Taiwan; 7 School of Medicine, China Medical University, Taichung, Taiwan

## Abstract

**Backgrounds:**

A common sequela of hip fracture is loss of ambulation capacity. Prediction of postoperative ambulation capacity is important for surgical and rehabilitation decision making. Handgrip strength is a quick and convenient tool for evaluating postoperative functional ability and outcome in variety of clinical conditions for the elderly and is associated with the use of walking aids. We propose that handgrip strength may be a good predictor for postoperative early ambulation. The purpose of our study was to investigate the contribution of handgrip strength in the prediction of postoperative early ambulation capacity in elderly hip fracture patients.

**Methods:**

Clinical data of patients with low-energy hip fractures who received surgery from Jan 2018 to Dec 2019 were prospectively collected. The correlations of ambulation time with complication rate, age, gender, injured side, fracture classifications, surgical procedure, body mass index (BMI), and handgrip strength were analyzed.

**Results:**

Sixty-three hip fracture patients were included in this study. Patients whose ambulation time was less than 3 days after the operation had significantly fewer postoperative complications (*P* = 0.006). Handgrip strength showed the strongest correlation with postoperative early ambulation capacity (*P* = 0.004). The handgrip strength threshold value for early ambulation was found to be 20.5 kg for male patients and 11.5 kg for female patients.

**Conclusion:**

Handgrip strength testis a quick and convenient tool for predicting postoperative early ambulation capacity. In elderly Asians, male patients with a handgrip strength above 20.5 kg and female patients with a handgrip strength above 11.5 kg suggest a high likelihood of early postoperative ambulation and a lower risk of complications after the hip surgery.

## Backgrounds

Despite advances in modern surgical techniques and instruments, hip fractures in the elderly still present a remarkable challenge for both physicians and patients. Hip fractures are associated with a mortality rate of 20–24 % in the first year after injury [1, 2]. Moreover, hip fracture is often followed by permanent restriction of independent daily living function. Around 20 % of hip fracture survivors require long-term nursing home care, and 40 % are unable to walk independently for the rest of their lives [3–6].

Defining the ambulation capacity of hip fracture patients after operation is crucial for optimizing postoperative rehabilitation as it enables clinicians to inform patients, plan rehabilitation courses, and obtain early information that is valuable for home adjustments after discharge from the hospital [7–10]. Predicting the timing of ambulation after the operation is also important. Patients with an ambulation time earlier than 3 days usually have fewer complications than those who need a longer time for ambulation [8].

Many predictors have been developed to predict the functional outcomes of hip fracture. Factors associated with unfavorable postoperative function include old age, multiple comorbidities, reduced pre-injury functional status, cognitive impairment, poor nutrition, poor social support, long hospital stay, and handgrip strength [11–13]. Among these, handgrip strength has been reported as a reliable predictor of short- and long-term functional outcomes, and has the advantage of being a quick and convenient test [14–16]. However, the association of handgrip strength and ambulation time during the perioperative period has not been reported in the literature.

Though muscle strength can be assessed at different body sites, handgrip strength can be easily measured and can serve as a strong surrogate measure of overall muscular strength [14, 17]. It has been reported to be a major factor associated with mobility, activities of daily living, fragility, propensity to fall, and hip fracture incidence [15, 18–20]. We proposed that handgrip strength can be predictor for early ambulation after hip fracture surgery. The purpose of this study was to investigate the association of handgrip strength with early postoperative ambulation capacity in hip fracture patients.

## Methods

### Patient enrollment

The medical and radiographic records of hip fracture patients who received surgery for hip fracture in our hospital between January 2018 and December 2019 were collected prospectively. The inclusion criteria were age 60 years or older and a low-energy trauma hip fracture. The exclusion criteria were pathologic fracture, previous fracture at the same site, and concurrent fracture of an upper limb, cognitive impairment (Mini Mental State Examination Test score < 23) and prevalent motor impairment due to neurologic diseases. Clinical data of patients who met the above criteria were collected and reviewed. Hip fractures were classified according to the AO/OTA classification system.

### Postoperative treatment and follow-up

Patients were allowed to start unrestricted weight-bearing motion exercises with a walker under close monitoring after the hip fracture surgery [21]. All of the operated hips were evaluated radiographically in the anteroposterior (AP) view and lateral view immediately after operation and every month thereafter until bone union was achieved.

### Treatment outcome assessments

Hand grip strength was measured with a Jarmar Hydraulic Hand Dynamometer using the Southampton protocol [22]. Subjects were seated with back support and the hips flexed as close to 90 degrees as could be tolerated. The subjects rested their forearms on the arms of the bed with the wrists in a neutral position. The measurer supported the weight of the device by resting it on his or her palm. Measurements were performed three times for each hand to give six readings in total. The best of the six grip strength measurements was used in the statistical analyses. Measurements were taken after adequate pain control had been achieved within 3 days after the operation [23, 24]. Early ambulation capacity was defined as the ability to walk with or without an assistive device for 10 meters before the third postoperative day [25]. Postoperative complication was defined as the appearance of one or more of the following conditions during the hospitalization: pneumonia, pressure ulcers, new-onset delirium, urinary tract infection, deep venous thrombosis, and pressure sore [26]. Factors considered to be potentially correlated with early ambulation including age, gender, injured side, fracture classifications, surgical procedure, duration of hospital stay, handgrip strength, and body mass index (BMI), were collected and analyzed.

### Statistical analysis

Data analysis was performed using SPSS software (Version 19.0; Chicago, Illinois). Univariate analysis was performed using frequencies for descriptive statistics. Chi-square and Fisher’s exact test were used for the analysis of categorical variables. Logistic regression was performed to evaluate the predictor of early ambulation after the hip fracture surgery. Receiver operating characteristic (ROC) curve analysis was used to assess the predictive capacity of handgrip strength on early ambulation. Youden index was used to find the maximal sum for the best cut-off point. Correlations were considered significant if p values were less than 0.05 (two-sided).

## Results

Sixty-three consecutive patients receiving operation for acute hip fracture were enrolled, including 31 patients with a femoral intertrochanteric fracture that was treated with open reduction and internal fixation and 32 patients with a femoral neck fracture that was treated with bipolar hemiarthroplasty or cannulated screws. There were 30 males (48 %) and 33 females (52 %) with a mean age at operation of 81 years (range 63 to 98 years). Among the 63 patients, 34 patients had not started walking at the end of inpatient rehabilitation. Twenty-nine patients were able to begin ambulation during the hospitalization. The mean ambulation time was 2.97 days after the operation (1–5 days). Postoperative complications included urinary infection in 1 patient, pressure sore in 8 patients, and new-onset delirium in 5 patients. None of these patients were in the early ambulation group (ambulation time < 3 days). Nineteen patients who had an ambulation time of less than 3 days after the operation had a significantly lower complication rate (0 %) than those who needed more time to begin ambulation (32 %)(*P* = 0.006) (Table 1).

**Table 1:** Relationship of postoperative complication and ambulation time

	All (*n* = 63)	Early ambulation (*n* = 19) (%)	Late ambulation (*n* = 44) (%)	*P* value
Complications				
No, n	49 (78)	19 (100)	30 (68)	**0.006**
Yes, n	14 (22)	0 (0)	14 (32)	

Factors that may contribute to early and late ambulation included age, gender, injured side, fracture type, implant type, BMI, and handgrip strength. Details of baseline characteristics according to ambulation time are shown in Table 2.

**Table 2:** Baseline characteristics of 63 hip fracture patients according to ambulation time

	All (*n* = 63)	Early ambulation (*n* = 19)	Late ambulation (*n* = 44)
Age, years			
Mean ± SD	81 ± 8.9	78 ± 9.5	83 ± 8.4
(Range)	(62–98)	(62–92)	(63–98)
Gender			
Male, n (%)	30 (48)	8 (42)	22 (50)
Female, n (%)	33 (52)	11(58)	22 (50)
Injured side			
Left, n (%)	31 (49)	19(47)	22 (50)
Right, n (%)	32 (51)	10(53)	22 (50)
Classification			
ITF, n (%)	31 (49)	8(42)	23 (52)
FNF, n (%)	32 (51)	11(58)	21 (48)
Operation			
ORIF	38 (60)	9 (47)	29 (66)
Hemiarthroplasty	25 (40)	10 (53)	15 (34)
Hospital stay	8.5 ± 4.67 (2–25)	7.3 ± 4.86 (3–25)	9.2 ± 4.47 (2–22)
Hand grip strength			
Mean ± SD	17 ± 6.6	21 ± 6.5	15 ± 5.9
(Range)	(2–32)	(8–32)	(2–26)
BMI			
Mean ± SD	23.2 ± 3.82	23.6 ± 4.72	23.0 ± 3.44
(Range)	(16.6–32.5)	(16.8–32.5)	(16.6–32.4)

*ITF* Intertrochanteric fracture, *FNF* Femoral neck fracture, *ORIF* Open reduction and internal fixation, *BMI* Body mass index

In the regression analysis, hand grip strength showed a statistically significant association with postoperative ambulation time after multivariate adjustment (*p* = 0.003) (Table 3).

**Table 3:** Multivariate regression of predictors for ambulation time > 3 days

Risk Factors	OR (95 % C.I.)	*P* value
Age (years)	1.01 (0.924 to 1.11)	0.777
Gender		
Male	1.00 (Ref.)	
Female	0.37 (0.04 to 3.02)	0.348
Injured Side		
Left	1.00 (Ref.)	
Right	1.65 (0.36 to 7.56)	0.517
Fracture type		
FNF	1.00 (Ref.)	
ITF	0.00 (0.00 )	0.999
Operation		
Hemiarthroplasty	1.00 (Ref.)	
ORIF	0.00 (0.00)	0.999
Handgrip strength	0.82 (0.68 to 0.98)	**0.003**
BMI	0.96 (0.68 to 1.07)	0.165

*ITF* Intertrochanteric fracture, *FNF* Femoral neck fracture, *ORIF* Open reduction and internal fixation, *BMI* Body mass index

**Fig. 1:**
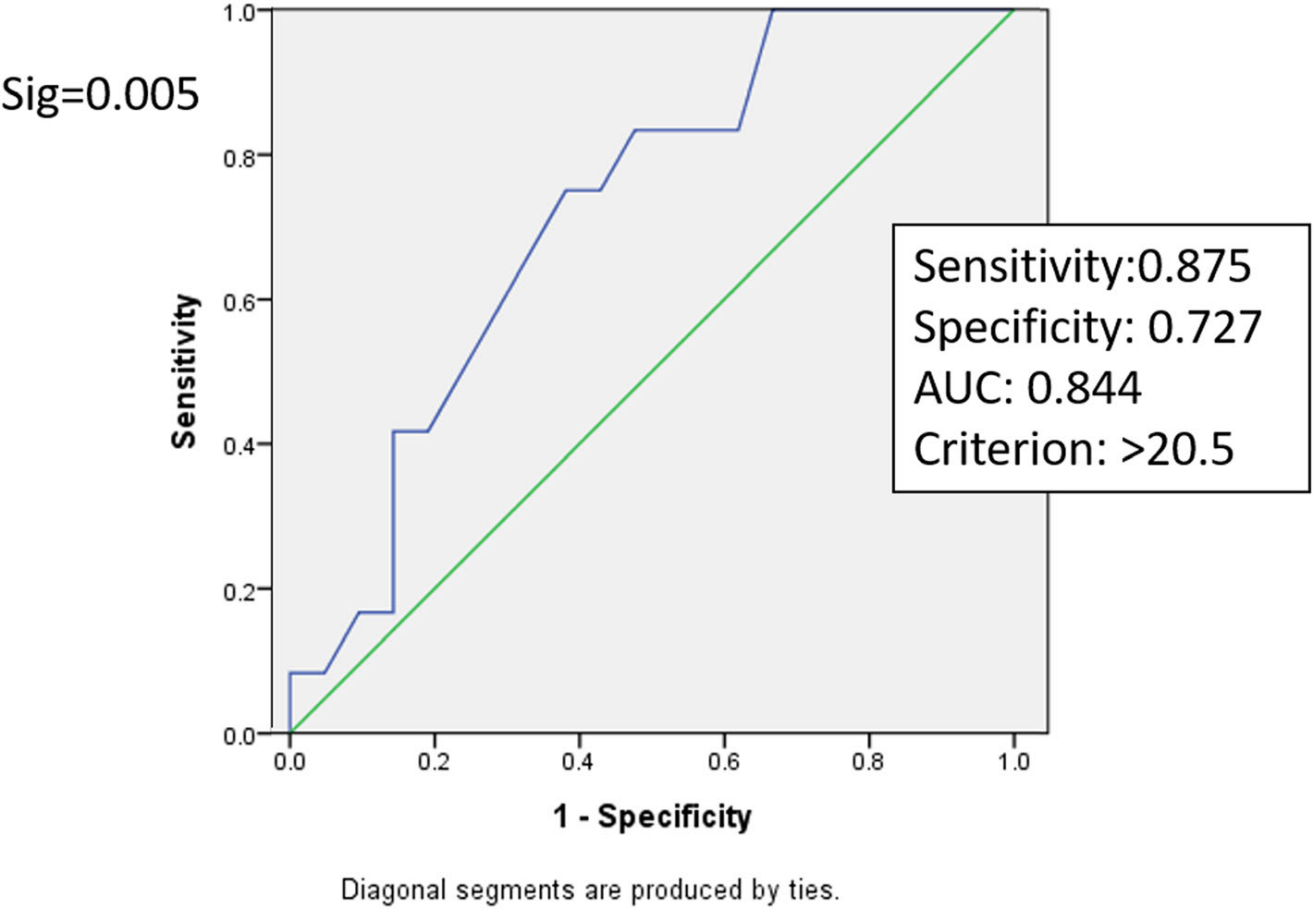
ROC Curve of the predictive model of early ambulation based on handgrip strength in female patients

**Fig. 2:**
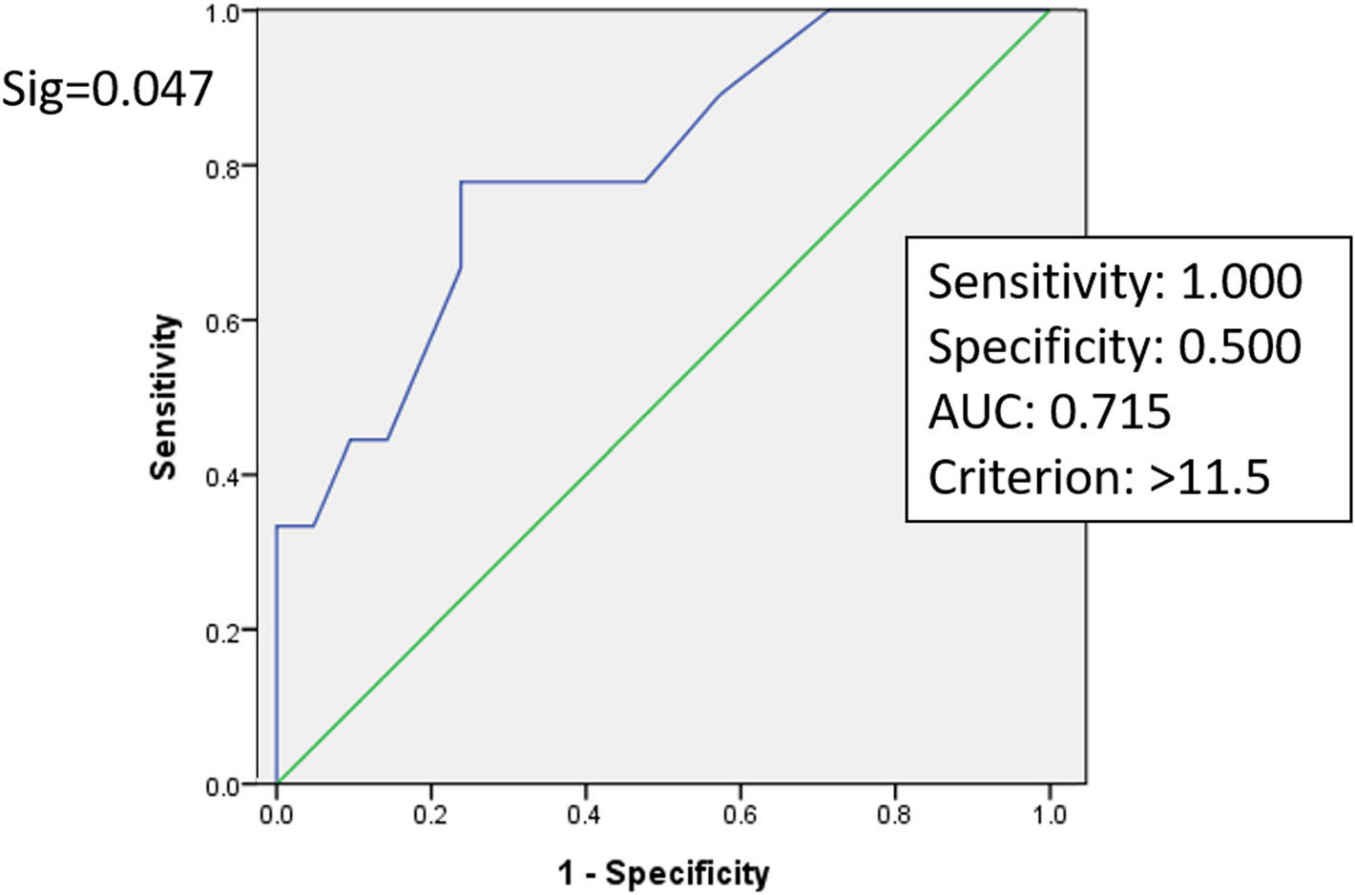
ROC Curve of the predictive model of early ambulation based on handgrip strength in male patients

ROC curve analysis was used to estimate a threshold value of hand grip strength that could predict early ambulation ability. For male patients, when handgrip strength was 20.5 kg, the sensitivity was 87.5 % and specificity was 72.7 %. The area under the curve (AUC) was 0.844 (Fig. 1), which was statistically significant (*p* = 0.005). For female patients, when handgrip strength was 11.5 kg, the sensitivity was 100 % and specificity was 50 %. The AUC was 0.715 (Fig. 2), which was statistically significant (*p* = 0.047).

## Discussion

Few studies have reported the relationship between handgrip strength and early postoperative ambulation capacity in the elderly with hip fracture. To the best of our knowledge, the present study is the first outcome-based investigation to define the optimal threshold of handgrip strength in order to predict postoperative ambulation capacity in senior Asians with hip fracture. We found that there was a significantly lower prevalence of postoperative complications in patients who could start ambulation earlier than postoperative day 3, and handgrip strength was the only significant factor capable of predicting postoperative early ambulation capacity. We also found that the minimum hand grip strength required to predict early postoperative ambulation capacity was 20.5 kg for men and 11.5 kg for woman.

Handgrip strength was reported to be an efficient and clinically relevant tool in predicting functional outcome in the elderly with hip fracture [14, 15]. Ji et al. found that grip strength was an independent predictor of ICU admission for hip fracture among the elderly. The combination of grip strength with red cell distribution width (RDS) even surpassed the ASA grade with respect to its ability to predict early postoperative complications in elderly hip fracture patients [15]. Di Monaco et al. reported that hand grip strength was strongly associated with Barthel index scores at the time of discharge and at the 6-month follow-up in women with hip fracture [14]. Our finding suggests that handgrip strength may be a useful tool to predict postoperative complication which is consistent with results reported by Ji et al. [15]. In addition to the predictive ability of short-term postoperative complications and long-term functional outcomes reported in previous reports, our study demonstrated that handgrip strength was also a strong predictive factor for ambulation capacity in the early postoperative period. Thus, we believe this result may have value in clinical practice as a simple and efficient tool for refining the decision-making process for rehabilitation course.

According to previously reported data, the factors associated with ambulation capacity after hip fracture include age, gender, prefracture ambulatory capacity and combined medical disease, cognitive status, serum albumin, serum folic acid, visual impairment, sarcopenia, and impaired communication [9, 27–30]. Though some of these factors were not included in our analysis, handgrip strength was reported to be an efficient parameter for assessing general comorbidities and cognitive status in the elderly [31].

There were several limitations in this study. First, the operations were not performed by a single surgeon. The operative skills of surgeons may have varied and this could have affected the treatment outcomes. Second, factors that may have confounded the effects of treatment method on outcomes, such as timing of measurement, cognitive function, arthritis of upper extremities, and laboratory data were not included in our statistical analysis. However, handgrip strength may have a valuable practical application in clinical practice as a surrogate marker of the general health status of the elderly. Third, the sample size of this study was relatively small and the study was carried-out in a single institution. Further multi-centered studies with a larger population should be done to confirm our findings.

## Conclusion

Handgrip strength is a simple and reliable tool to predict early ambulation capacity after hip fracture operations. Males with a handgrip strength more than 20.5 kg and females with handgrip strength more than 11.5 kg indicated a high likelihood of early ambulation after operation.

## Data Availability

The datasets used and analyzed during the current study are available from the corresponding author on reasonable request.

## Abbreviations

ORIF: Open reduction and internal fixation
ASA: American Society of Anesthesiologists
AO/OTA: Arbeitsgemeinschaft für Osteosynthesefragen/ Orthopaedic Trauma Association
AP: Anteroposterior
BMI: Body mass index
